# Pandemônio Durante a Pandemia: Qual o Papel dos Profissionais da Saúde e a Ciência?

**DOI:** 10.36660/abc.20200320

**Published:** 2020-05-22

**Authors:** André d’Avila, Marco F. Vidal Melo, Renato D. Lopes

**Affiliations:** 1 Serviço de Arritmia Cardíaca Hospital SOS Cardio FlorianópolisSC Brasil Serviço de Arritmia Cardíaca, Hospital SOS Cardio, Florianópolis, SC – Brasil; 2 Critical Care and Pain Medicine Massachusetts General Hospital Harvard Medical School BostonMA USA Department of Anesthesia, Critical Care and Pain Medicine, Massachusetts General Hospital, Harvard Medical School, Boston, MA – USA; 3 Duke Clinical Research Institute Duke University School of Medicine DurhamNC USA Division of Cardiology, Duke Clinical Research Institute, Duke University School of Medicine, Durham, NC – USA

**Keywords:** Coronavirus, COVID-19, Pandemias, Hydroxychloroquina/efeitos da medicação, Azitromicina/efeitos adversos, Aprovação de Drogas, Comitê de Ética em Pesquisa, Mídias Sociais

A conduta dos comentaristas esportivos brasileiros durante a recente pandemia da COVID-19 merece destaque. Dia após dia, mesmo socialmente distantes, eles vêm à televisão dar a sua contribuição para amenizar o desconforto do público. Durante horas, discutem exaustivamente jogos do passado, problemas atuais e o futuro do esporte sobre o qual se especializaram. Em nenhum momento, no entanto, se arriscam a comentários fora da sua área. Mesmo que, como cidadãos, tenham uma opinião sobre a gravidade da COVID-19, sobre o benefício do distanciamento social ou sobre o uso de medicamentos sem comprovação científica, preferem continuar restritos às áreas de excelência nas quais são reconhecidos como especialistas, pois conhecem aquele assunto melhor que ninguém.

Mais difícil de compreender, entretanto, é a postura dos que insistem em emitir opinião não fundamentada sobre várias intervenções no tratamento da COVID-19, mesmo sabendo que não há confirmação baseada em alto nível de evidência do potencial efeito benéfico dessas recomendações. Alguns simplesmente optam pelo proselitismo médico. Outros, por não reconhecerem nem entenderem a necessidade da investigação clínica adequada, preferem a omissão: não aprovam nem desaprovam nada, deixando a população sem nenhum referencial.^1^ Contudo, o tipo de atitude mais comum talvez seja a do médico assustado pelo desconhecido, desesperado por seus entes queridos e pacientes, pressionado por respostas e influenciado pelo bombardeio das informações nas mídias sociais e que, por isso, agarra-se à possibilidade de um tratamento redentor. Infelizmente, a única doença tratada com essa abordagem costuma ser a ansiedade dos próprios médicos e seus pacientes.

Dentro desse panorama desolador de incertezas, informações desencontradas e ausência de lideranças, a torrente de asserções com início e eco nas inúmeras mídias sociais transforma em verdade (ou pelo menos em expectativa) tudo o que é divulgado em primeira mão, por mais absurdo que seja. O único interesse é a notícia em si. As consequências não importam. Entretanto, ao desprezar o método científico rigoroso, com o álibi de tentar ajudar, criam um ambiente de confusão – ou pior, um ambiente que aumenta o risco daqueles que tomam tais asserções como verdadeiras.

O rigor científico e ético da pesquisa como um todo, e na área médica em especial, beneficiou milhares de pacientes em todo o mundo por meio da pesquisa criteriosa, sem atalhos, realizada sob a orientação cuidadosa da Declaração de Helsinki de 1964. O princípio básico da Declaração é o respeito ao indivíduo que deve consentir sua participação e permanência no protocolo de pesquisa, visto que seus interesses precedem os interesses da ciência e da sociedade. No entanto, se o interesse do paciente se impõe aos interesses científicos, como justificar a necessidade de randomizar alguém que possa eventualmente ter o “azar” de ser alocado no grupo controle, talvez placebo, se no outro extremo existe, ou pelo menos se supõe que exista, esperança de cura?

A resposta a essa pergunta deriva da aceitação do método científico: somente a demonstração objetiva e quantificável, acima de mera casualidade da eficácia e efetividade de qualquer intervenção, merece aceitação, ainda que várias ideias pareçam viáveis e eficazes em etapas preliminares. A existência do grupo controle é essencial nesse aspecto. Este deve oferecer ao participante do ensaio clínico uma relação benefício/risco presumivelmente equivalente (“equipoise”) àquela da intervenção baseada nas evidências disponíveis quando da implementação do estudo. Assim, uma medida objetiva de comparabilidade da intervenção é imposta e deve ser vencida. Além disso, sabe-se que pacientes que participam de ensaios clínicos evoluem melhor que aqueles que não entram em tais pesquisas, mesmo quando alocados ao grupo controle ou placebo. Lembremos, portanto, que a maneira mais segura de tratar um paciente quando não temos a resposta é incluí-lo em um ensaio clínico, pois somente assim haverá o melhor tratamento possível, sob supervisão direta, enquanto ajuda o avanço da ciência. A história da medicina é farta de exemplos de tratamentos considerados “absolutamente efetivos” que mostraram-se fúteis ou até maléficos após a realização dos ensaios clínicos randomizados, considerado o padrão-ouro para se definir eficácia e segurança de qualquer intervenção em medicina.

Na cardiologia, casos de futilidade e malefícios são inúmeros e gritantes. O uso de fármacos antiarrítmicos para prevenção de morte súbita em pacientes com extrassístoles ventriculares após infarto agudo do miocárdio (IAM), de magnésio para redução da área infartada e de betabloqueador na síncope vasovagal consiste em alguns exemplos da enorme diferença entre a expectativa (ou o senso comum) e o real efeito de intervenções terapêuticas. Sem esses estudos, milhares de pacientes teriam sido submetidos a procedimentos ineficazes, dos quais, em vez de benefícios, derivariam apenas complicações.

Na situação atual de enfrentamento da COVID-19, vale a pena mencionar que todas as alternativas supostamente milagrosas (desde o uso de doses maciças de vitamina C, D e zinco até o uso de macrolídios, cloroquina e derivados, corticosteroides, antivirais e outras medicações) já foram testadas em outras viroses e epidemias, tais como HIV, Ebola e H1N1. Infelizmente, apesar da expectativa inicial, não se mostraram seguras ou eficazes nesses ensaios anteriores.

Naturalmente, o efeito de algumas dessas intervenções pode ser diferente na pandemia atual. Tal hipótese, no entanto, precisa ser avaliada e provada com todo o rigor científico que a urgência e a gravidade da situação impõem. Infelizmente, a maioria dos trabalhos iniciais utilizados para basear a indicação de diversas intervenções no combate à COVID-19 é um exemplo de pseudoevidência. São cientificamente risíveis.

Tomemos como exemplo o estudo que popularizou a hidroxicloroquina^1^ (aquele citado por Donald Trump como tendo “probabilidade de ser uma das maiores descobertas da história da medicina”). Os autores do trabalho investigavam se pacientes com COVID-19 teriam melhor desfecho com a hidroxicloroquina. Para tanto, teria sido mandatório que, de dois grupos semelhantes de pacientes, apenas um tivesse recebido o fármaco. Parece elementar, mas não foi isso que aconteceu: além do remédio diferente, os grupos eram de hospitais distintos, tinham idades diversas, apresentavam condições clínicas e tratamentos variados, além de cargas virais diferentes. Assim, como isolar o efeito do remédio? Além disso, os quatro pacientes que morreram ou foram à unidade de terapia intensiva (UTI) – aliás, todos tomando hidroxicloroquina – foram eliminados dos resultados, o que sugere que, para alguns, um óbito possa ser menos relevante do que a detecção de vírus na nasofaringe. Além disso, o tamanho da amostra, queixa comum de vários revisores, foi muito pequeno, não permitindo definir qualquer possível efeito de tratamento.

É decepcionante quando um estudo não consegue responder à pergunta proposta. É pior quando isso gera uma convulsão social. O mais dramático, no entanto, é saber que tal artigo passou por uma revisão de pares e por um editor que, se tivesse agido de forma responsável, abrindo mão do frenesi do aumento do número de acessos ao *website* da revista e da notoriedade imediata, poderia ter evitado essas consequências em bola de neve. Uma pandemia não justifica o esquecimento da ciência e os erros que criam falsas esperanças que potencialmente coloquem vidas em risco.

A atitude de médicos e cientistas no mundo varia com relação à interpretação desses dados. Muitos acham que aquela evidência sobre o uso da cloroquina justifica a prescrição do medicamento, mas essa posição está longe de ser unânime. Vários colegas médicos que contraíram a COVID-19 aceitaram participar de estudos clínicos randomizados, contribuindo para que uma resposta metodologicamente adequada fosse alcançada, com potencial benefício a milhares de pessoas. Nesse sentido, para esta e outras eventuais pandemias, vale lembrar que somente estudos clínicos rigorosos realizados com a colaboração de profissionais, hospitais e sociedades médicas do mundo todo, e liderados por especialistas em pesquisa clínica, podem oferecer as respostas corretas, nos livrando da mazela do curandeiro, que capitaliza o sucesso da eventual melhora e, no caso de um desfecho inadequado, sugere que o paciente não foi merecedor da dádiva por ele oferecida.

Médicos, outros profissionais da saúde, cientistas e as sociedades que os representam têm o dever de nos livrar do retrocesso que o não entendimento do método científico acarreta, além da obrigação de nos lembrar de que o senso comum erra e que nosso estado de humor coletivo não pode justificar deslizes metodológicos que possam impactar milhares de vidas. Espera-se que os médicos façam o que sabem fazer de melhor: agir à luz da ética, de forma pragmática, com base no melhor que a ciência pode oferecer. Sejamos realmente especialistas quando os dados de alta qualidade científica estiverem disponíveis. Afinal, a verdade sempre prevalece, e a ciência é a ferramenta que mais rapidamente nos aproxima dela. Como médicos e cientistas, nosso papel é encurtar o caminho entre a hipótese e a conclusão fundamentada, tanto para benefício dos pacientes sob nossa responsabilidade quanto para a população que anseia por uma resposta segura da ciência médica.
