# Supplementary material for: Pandemônio Durante a Pandemia: Qual o Papel dos Profissionais da Saúde e a Ciência?
Source: Arq Bras Cardiol. 2020 May 22;114(5):753–4. [Article in Portuguese] doi: 10.36660/abc.20200320 (PMC8386997; doi:10.36660/abc.20200320)
Supplement: Translation

# Translation: Pandemonium During the Pandemic: What is the Role of Health and Science Professionals?

**Authors:** André d’Avila, Marco F. Vidal Melo, Renato D. Lopes

**Affiliations:** 1 Brazil Serviço de Arritmia Cardíaca, Hospital SOS Cardio, Florianópolis, SC – Brazil; 2 USA Department of Anesthesia, Critical Care and Pain Medicine, Massachusetts General Hospital, Harvard Medical School, Boston, MA – USA; 3 USA Division of Cardiology, Duke Clinical Research Institute, Duke University School of Medicine,Durham, NC – USA

**Keywords:** Coronavirus, COVID-19, Pandemics, Hydroxychloroquine/effects medication, Azythromicine/adverse effects, Drug Approval, Ethics Committees, Research, Social Media

The behavior of Brazilian sports commentators during the recent COVID-19 pandemic is noteworthy. Day after day, despite social distancing, they continued their television broadcasts to help ease their viewers’ discomfort. For hours, they discussed past games, current problems and the future of the sport. At no time, however, did they make comments on topics outside of their area of expertise. Although they most certainly had an opinion on the seriousness of COVID-19, the merits of social distancing or use of medications popularized by many without scientific evidence, they restricted their commentary to their areas of expertise – sports!

What has been hard to understand is the insistence of some physicians to provide opinions on unsubstantiated interventions for the treatment of COVID-19. Some simply choose medical proselytism. Others may not appreciate the need for proper scientific investigation. Many prefer to omit themselves from any debate. However, the most perplexing type of attitude is simply a reactionary behavior related to the fear of the unknown that is, the combination of desperation and need to provide care for patients, and bombardment of information from social media may bias many doctors to embrace any potential redemptive treatment. Unfortunately, the only disease treated with this approach is the anxiety shared by doctors and their patients.

In this bleak scenario full of uncertainties, mismatched information, lack of leadership, and a raging torrent of assertions in social media can transform claims into truth (or at least into expectations), however absurd they may be. Unfortunately, disregarding strict scientific methods, using an alibi of trying to help, creates an atmosphere of confusion and increases the risk of those who implement such statements.

The scientific and ethical rigor of research as a whole, and particularly in the medical field, has benefited thousands of patients around the world through careful research carried out under the guidance of the Declaration of Helsinki from 1964. The basic principle of the Declaration is respect for the individuals (who must consent to participate in the research protocol), as the individual’s interests precede that of science and society. However, if the patient’s interest is imposed then one logically will ask how is it possible to justify randomization given the fears patients or physicians may have with the possibility of having “bad luck” and being allocated to a control group, perhaps a placebo, when the alternative is perceived to be a hope for cure?

The answer to this question requires understanding and acceptance of the scientific method. Although several ideas appear effective during preliminary stages, only objective demonstration of efficacy beyond mere chance merits acceptance. In that regard, a control group is indispensable to achieve this as it allows determination of the benefit/risk ratio (“equipoise”) of the intervention. While participation in a control group may be met with disappointment from some patients and physicians, it must be remembered that individuals who participate in clinical trials in general do better than those who do not participate in such studies, even when allocated to control or placebo groups. Thus a logical and safe way to treat a patient when an answer to a clinical question is not available is to include them in a clinical trial as these patients will be offered the best possible treatment, under direct supervision, while advancing science.

The history of medicine is full of examples of treatments considered by experts as “absolutely effective” that clinical trials proved to be futile and even harmful. In cardiology, cases of futility and harm are numerous and even striking. The use of antiarrhythmic drugs to prevent sudden death in patients with ventricular extrasystoles after acute myocardial infarction (AMI), magnesium to reduce the infarcted area and beta-blockers in vasovagal syncope are examples of the huge difference between expectation (perceived “common sense”) and the actual effect resulting in a therapeutic upheaval.

In the current situation of COVID-19, supposedly miraculous therapies (including supratherapeutic doses of vitamins [C, D, and zinc], macrolides, chloroquine and its derivatives, corticosteroids, antivirals and other medications) have been tested in clinical trials for other viruses including HIV, Ebola and H1N1 and, despite the expectations of efficacy in these conditions, none were shown to be safe or effective. While it may be assumed that the effect of some of these interventions might work differently in the current Covid pandemic, these beliefs will need to be evaluated with scientific rigor that the urgency and gravity of the situation entails.

Unfortunately, most recommendations of interventions to fight COVID-19 are based in pseudo-evidence. The study that popularized hydroxychloroquine^1^ (*the one cited by Donald Trump as having “a real chance to be one of the biggest game changers in the history of medicine”*) is scientifically ludicrous. The authors of the study investigated whether patients with COVID-19 would have a better outcome with hydroxychloroquine. To that end, it would have been mandatory that two similar groups of patients would exist with only one receiving the drug. As simple as this seems, that is not what happened. In addition to the different drugs administered, the groups were from different hospitals, were of different ages, had different clinical conditions and received different additional treatments. Most notably, these patients had different viral loads. How is one to isolate the effect hydroxychloroquine when such marked differences are present? On top of that, four patients who died or went to the intensive care unit (ICU) who received hydroxychloroquine were eliminated from the results suggesting that for some investigators death may be less relevant than the detection of a virus in the nasopharynx. Finally, the sample size was very small not allowing any possible effect from the treatment to be defined.

While it is disappointing when a study fails to answer the proposed question, it is worse when it creates social upheaval. This article was peer-reviewed by colleagues and by an editor who could have avoided consequences of this publication had they acted responsibly. A pandemic does not justify forgetting science as mistakes create false hopes that may potentially put lives at risk.

There has been variable interpretation of these data by the medical community throughout the world. Whereas many believe that the use of chloroquine is justifiable, that stance is far from unanimous. Many health care workers diagnosed with COVID-19 agreed to participate in randomized clinical trials to help create high-quality data that may potentially benefit thousands of people. It is remarkable that the medical community would band together as subjects in a clinical trial to generate data for a disease they are helping the public fight! This is the correct decision. Only properly designed and executed clinical studies conducted by professionals, hospitals and medical societies globally, and led by experts in clinical research, can offer accurate answers. The medical fraternity has a duty to free us from the setbacks created by the failure to understand scientific methods. “Common sense” and our collective mood cannot justify methodological errors which in turn may adversely impact thousands of lives. Physicians are expected to do what they best do - act in the light of ethics, pragmatically, based on the best that science can offer. Let us be genuine specialists when high-quality scientific data is available. After all, truth always prevails, and science is the tool that most rapidly draws us closer to it. As doctors and scientists, our role is to abate the gap between assumptions and reasoned conclusions as this will benefit patients and the population that yearns for answers provided by medical science.
